# Correction: Almessiere et al. Correlation Between Composition and Electrodynamics Properties in Nanocomposites Based on Hard/Soft Ferrimagnetics with Strong Exchange Coupling. *Nanomaterials* 2019, *9*, 202

**DOI:** 10.3390/nano16140875

**Published:** 2026-07-16

**Authors:** Munirah Abdullah Almessiere, Alex V. Trukhanov, Yassine Slimani, K. Y. You, Sergei V. Trukhanov, Ekaterina L. Trukhanova, F. Esa, A. Sadaqat, K. Chaudhary, Maxim Zdorovets, Abdulhadi Baykal

**Affiliations:** 1 Department of Physics, College of Science, Institute for Research & Medical Consultations (IRMC), Imam Abdulrahman Bin Faisal University, P.O. Box 1982, Dammam 31441, Saudi Arabia; malmessiere@iau.edu.sa; 2 Department of Nano-Medicine Research, Institute for Research & Medical Consultations (IRMC), Imam Abdulrahman Bin Faisal University, P.O. Box 1982, Dammam 31441, Saudi Arabia; abaykal@iau.edu.sa; 3 SSPA “Scientific and Practical Materials Research Center of NAS of Belarus”, 220072 Minsk, Belarus; sv_truhanov@mail.ru (S.V.T.); kastor1986@yandex.ru (E.L.T.); 4 South Ural State University, 454080 Chelyabinsk, Russia; 5 National University of Science and Technology MISiS, 119049 Moscow, Russia; 6 Department of Physics Research, Institute for Research & Medical Consultations (IRMC), Imam Abdulrahman Bin Faisal University, P.O. Box 1982, Dammam 31441, Saudi Arabia; yaslimani@iau.edu.sa; 7 School of Electrical Engineering, Faculty of Engineering, Universiti Teknologi Malaysia, Skudai-Johor 81310, Malaysia; kyyou@fke.utm.my; 8 Physics and Chemistry Department, Faculty of Applied Sciences and Technology, Universiti Tun Hussein Onn Malaysia, Pagoh-Johor 81310, Malaysia; fahmir@uthm.edu.my; 9 Mechanical and Energy Engineering Department, College of Engineering, Imam Abdulrahman Bin Faisal University, P.O. Box 1982, Dammam 31441, Saudi Arabia; truhanov86@gmail.com; 10 Physics Department, Faculty of Science, Univerity Teknology Malaysia, Johor Bahru-Johor 81310, Malaysia; kashif@utm.my; 11 L.N. Gumilyov Eurasian National University, Astana 10008, Kazakhstan; m_zdorovets@mail.ru; 12 The Institute of Nuclear Physics of Republic of Kazakhstan, Astana 10008, Kazakhstan; 13 Ural Federal University Named After the First President of Russia B.N. Yeltsin, 620002 Yekaterinburg, Russia

## Figure/Table Legend

In the original publication [1], a previously published article [30] was cited two times in the manuscript. Unfortunately, the authors regret the oversight of not properly citing this reference in the captions of Table 1, Figure 1, and Figure 3, which contained data reused from the previous study (reference [30]).

The citation has now been inserted in Section “3.1. Crystal Structure and Microstructure”, and the legends should now read as follows:

**Figure 1.** The X-ray diffraction powder patterns of the Sr_0.3_Ba_0.4_Pb_0.3_Fe_12_O_19_/(CuFe_2_O_4_)_x_ composite samples x = 2 (**a**), 3 (**b**), 4 (**c**), and 5 (**d**). Images were reused with permission from [30] (copyright 2018, Elsevier).

**Table 1.** The features of the crystal structure (a and c unit cell parameters and D—average crystallite size) for each phase (Sr_0.3_Ba_0.4_Pb_0.3_Fe_12_O_19_ and CuFe_2_O_4_) of composites were obtained using XRD data. The data presented in this table were reused with permission from [30] (copyright 2018, Elsevier).

**Figure 3.** HRTEM images of S_r0.3_Ba_0.4_Pb_0.3_Fe_12_O_19_/(CuFe_2_O_4_)_x_ composite samples. Images were reused with permission from [30] (copyright 2018, Elsevier).

## Text Correction

There was also a minor error in the original publication [1] regarding the SEM instrument used. The correct SEM model was the “FEI Teneo” instead of the “Hitachi S-4800”. A correction has been made to Section “2. Materials and Methods” in the first paragraph as follows:

“The peculiarities of the chemical composition and microstructure were analyzed using Scanning Electron Microscopy (SEM; FEI Teneo, Hillsboro, OR, USA) with Energy-dispersive X-ray spectroscopy (EDX).”

The authors state that the scientific conclusions are unaffected. This correction was approved by the Academic Editor. The original publication has also been updated.
